# Prognostic and Clinicopathological Significance of Circular RNA circ-ITCH Expression in Cancer Patients: A Meta-analysis

**DOI:** 10.1155/2021/8828299

**Published:** 2021-02-02

**Authors:** Xiao-Dong Sun, Chen Huan, Da-Wei Sun, Guo-Yue Lv

**Affiliations:** ^1^Department of Hepatobiliary and Pancreatic Surgery, The First Hospital of Jilin University, Changchun, 130021 Jilin Province, China; ^2^Institute of Virology and AIDS Research, The First Hospital of Jilin University, Changchun, 130021 Jilin Province, China

## Abstract

Circular RNAs are a class of RNAs with a covalently closed configuration, and several members of them have been reported to be capable of regulating various biological processes and predicting the outcome of disease. Among them, circular RNA circ-ITCH has been identified to be aberrantly expressed and associated with disease progression in diverse cancers. However, the correlation of circ-ITCH expression with clinicopathological features, as well as the prognosis of cancers, remains inconclusive. Therefore, a meta-analysis was performed to investigate the clinical significance of circ-ITCH in cancers by systematically summarizing all eligible literatures. Up to August 31, 2020, relevant articles were searched in PubMed, Web of Science, Cochrane library, Embase, CNKI, and Wanfang databases. Pooled hazard ratios (HRs) and odds ratios (ORs) with corresponding 95% confidence intervals (CIs) were calculated. A total of 1604 patients from 14 studies were included in this meta-analysis. The results showed that cancer patients with low circ-ITCH expression were more susceptible to develop lymph node metastasis (OR = 2.25, 95% CI: 1.67-3.02, *p* ≤ 0.01), larger tumor size (OR = 3.01, 95% CI: 2.01-4.52, *p* ≤ 0.01), advanced TNM stage (OR = 2.82, 95% CI: 1.92-4.14, *p* ≤ 0.01), and poor overall survival (OS) (HR = 2.45, 95% CI: 2.07–2.90, *p* ≤ 0.01, univariate analysis; HR = 2.69, 95% CI: 1.82-3.96, *p* ≤ 0.01, multivariate analysis). Thus, low circ-ITCH expression was significantly associated with aggressive clinicopathological features and unfavorable outcome in various cancers. Therefore, circ-ITCH may serve as a molecular therapy target and a prognostic marker in human cancers.

## 1. Introduction

Circular RNAs (circRNAs) are a class of newly discovered RNAs with a covalently closed configuration that exist in various organisms [1]. circRNAs are generated from precursor mRNAs (pre-mRNAs) via the back-splicing of exons, introns, or both, to form a covalently closed continuous loop with no 5′ caps and 3′ poly (A) tails [2]. Initially, circRNAs were considered byproducts of splicing errors. With the advances in the field of high-throughput sequencing, an increasing number of circRNAs and their functions have been characterized [3]. Functionally, circRNAs exert their regulation role through multiple mechanisms. Some circRNAs can work as competing endogenous RNA (ceRNA) to sequester miRNAs and reduce their activity, which in turn positively regulates the expression of miRNA-related target genes [4]. Besides, several circRNAs have been revealed to bind to target proteins by acting as protein decoys [5]. Also, circRNAs can regulate the transcription of targeted genes through interacting with transcription factors [6]. Importantly, recent reports have proved that a subset of circRNAs can be translated [7]. In particular, the essential role of circRNAs in cancer development has been illustrated. Several circRNAs have been reported to be aberrantly expressed and exert oncogenic or tumor-suppressor function in cancers, possibly by acting as sponges for miRNAs. For instance, one of the earliest and best-characterized circRNAs, ciRS-7, contains more than 70 binding sites for miR-7 [2]. Therefore, ciRS-7 was proved to promote cancer progression by acting as miR-7 sponges to upregulate the direct target genes of miR-7 and activate cancer-related pathways. ciRS-7 was found to facilitate the more aggressive phenotype of gastric cancer via suppressing the miR-7-mediated PTEN/PI3K/AKT signaling pathway [8]; in colorectal carcinoma, ciRS-7 was reported to activate the EGFR/RAF1/MAPK pathway via antagonizing miR-7 activity [9]; the ciRS-7/miR-7/NF-*κ*B axis was demonstrated to play a crucial role in accelerating development of esophageal squamous cell carcinoma and lung cancer [10, 11]. circRNAs are also implicated in angiogenesis of cancers. circRNA-MYLK was found to activate vascular endothelial growth factor A (VEGFA) through acting as a sponge of miR-29a, thereby promoting tumor growth, metastasis, and angiogenesis of bladder cancer [12]. Furthermore, circRNAs were implicated in cancer therapy as well. Zhu et al. reported that knockdown of circPVT1, which was found upregulated in osteosarcoma (OS) tissues and chemoresistant cell lines, impairs the resistance to doxorubicin and cisplatin of OS cells a of classical drug resistance-related gene ABCB1 [13]. Furthermore, significant association between the expression of circRNAs and the progression of cancers has been found, indicating the potential of circRNAs to serve as a biomarker for predicting the outcome of cancers [14–17].

Recently, circular RNA Itchy E3 ubiquitin protein ligase (circ-ITCH), a novel circular RNA originated from exons of gene itchy E3 ubiquitin protein ligase (ITCH), located on chromosome 20q11.22, was reported to be lower expressed in several cancers [18]. So far, circ-ITCH has been proved to be implicated in prostate cancer [19, 20], ovarian cancer [21–24], bladder cancer [25], breast cancer [26], lung cancer [27], oral squamous cell carcinoma [28], gastric cancer [29], hepatocellular carcinoma [30], glioma [31], and multiple myeloma [32]. Accumulating evidence has implied that circ-ITCH exert tumor-suppressor function in these cancers by acting as a sponge for oncogenic microRNAs. Although the correlation between circ-ITCH expression and cancer progression has been investigated by these studies above, most individual studies have been limited by inconsistent conclusions or small sample sizes. Thus, we performed this quantitative meta-analysis by systematically evaluating the relationship between circ-ITCH expression and the clinicopathological parameters as well as prognosis of cancers with all eligible articles.

## 2. Materials and Methods

### 2.1. Publication Search

Our literature search was performed following the preferred reporting items for systemic reviews and meta-analyses (PRISMA) statement criteria [33]. A comprehensive electronic search was performed in PubMed, Web of Science, Cochrane library, Embase, CNKI, and Wanfang databases updated to August 31, 2020. The keywords during the literature search were “circular RNA ITCH” or “circ-ITCH” or “cir-ITCH” or “circular RNA Itchy E3 ubiquitin protein ligase”. The search strategy in PubMed was “*circular RNA ITCH [All Fields] OR circ-ITCH [All Fields] OR cir-ITCH [All Fields] OR circular RNA Itchy E3 ubiquitin protein ligase [All Fields]*.” The search strategy in Web of Science was *“TS = (circular RNA ITCH OR circ-ITCH OR cir-ITCH OR circular RNA Itchy E3 ubiquitin protein ligase)*”. In addition, the citation lists of retrieved articles were screened manually for potential eligible studies.

### 2.2. Inclusion and Exclusion Criteria

Studies were considered eligible if they fulfilled the inclusion criteria as follows: (1) articles investigated the correlation of circ-ITCH expression with cancer progression and/or clinicopathological factors, (2) the expression of circ-ITCH in cancerous tissues was measured, (3) cancer patients were divided into high/low groups according to the circ-ITCH expression, and (4) related clinicopathological parameters and/or prognostic results were described. Exclusion criteria of the present meta-analysis are the following: (1) duplicated publications; (2) reviews, letters, comments, and conference articles; (3) articles irrelevant to the present study; or (4) studies without available data.

### 2.3. Data Extract

Two investigators (Xiao-Dong Sun and Chen Huan) performed the data extraction from the eligible studies independently. Discrepancies were resolved by discussion with a third investigator (Da-Wei Sun) to reach a consensus. The following items were collected from each included study: first author, year of publication, origin of patients, cancer type, number of patients, detecting method of circ-ITCH expression, cutoff value for grouping, number of patients with lymph node metastasis (LNM), larger tumor size and advanced TNM stage in each group, follow-up period, survival analytical method (multivariate or univariate), and hazard ratio (HR) with 95% confidence interval (CI) for overall survival (OS). When the prognosis was plotted as a Kaplan-Meier curve, the software Engauge Digitizer version 4.1 (http://digieizer.sourceforge.net/) was applied to digitize the data, and HR with 95% CI was calculated as described [34].

### 2.4. Quality Assessment

The methodological quality of the included studies was assessed with Newcastle-Ottawa Scale (NOS) criteria, which is scored based on subject selection, comparability of subject, and clinical outcome [35]. The final scores of NOS ranged from 0 to 9, and studies with scores ≥ 6 were considered to be of high quality.

### 2.5. Data Analysis

Meta-analyses were conducted using Stata SE12.0 (Stata Corporation, College Station, Texas). Pooled HR > 1 indicated that low circ-ITCH correlated with poor prognosis, and pooled odds ratio (OR) > 1 indicated low circ-ITCH correlated with poor clinicopathological outcomes. The heterogeneity among the included studies was assessed through *χ*^2^-based *Q* test and *I*^2^ statistics. When the *I*^2^ value > 50% and/or *p* < 0.10, indicating that the heterogeneity was significant, a random-effects model was applied; otherwise, the fixed-effects model was adopted. To assess the publication bias of included studies, Begg's and Egger's tests were conducted to estimate the potential publication bias quantificationally, where a *p* value < 0.05 illustrates significant publication bias [36, 37]. In addition, funnel plots were used to present the distribution of included studies' results when more than 8 studies were included, which represented the publication bias based on visual inspection. Sensitivity analysis was also performed to evaluate the effect of each individual study on the overall effect of meta-analysis results. All tests were two-sided; *p* values < 0.05 were considered statistically significant.

## 3. Results

### 3.1. Literature Information

**Figure 1:**
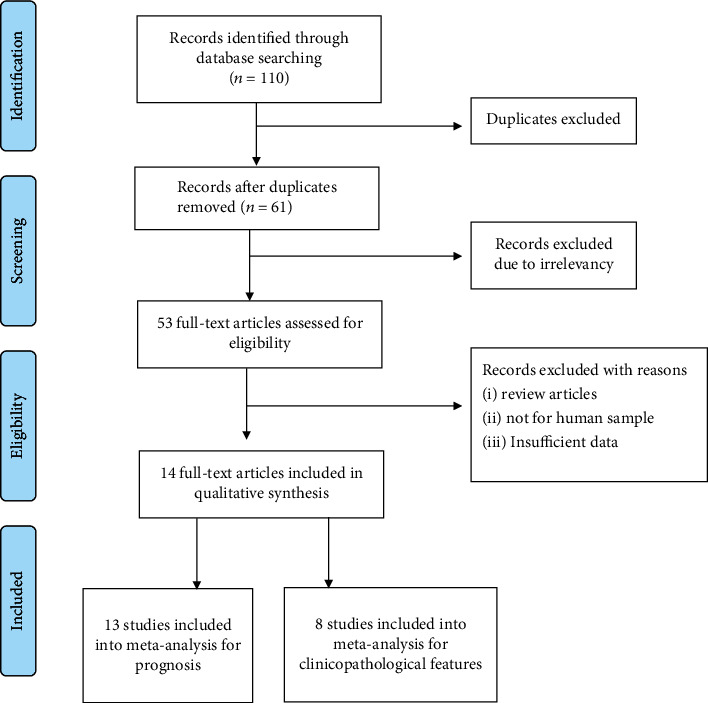
Literature selection process by following PRISMA guidelines in this meta-analysis.

The flow diagram for literature screening and selection was shown in Figure 1. A total of 110 records were retrieved by searching the databases, and 96 articles were excluded according to the inclusion and exclusion criteria. Finally, 14 articles comprising 1604 patients were identified as eligible and included in the present meta-analysis.

### 3.2. Study Characteristics

The main characteristics of eligible studies are summarized in Table 1. These 14 enrolled articles were published between 2017 and 2020 with sample sizes ranging from 20 to 288. Most of the populations were from China and divided into a high or low group based on the median value of circ-ITCH expression. The expression of circ-ITCH was detected with the method of quantitative reverse transcription-polymerase chain reaction (qRT-PCR) in all 14 populations. According to the NOS criteria, all of the included studies got scores ≥ 6, indicating their high methodological quality.

### 3.3. Association between circ-ITCH and Clinicopathological Parameters

**Figure 2:**
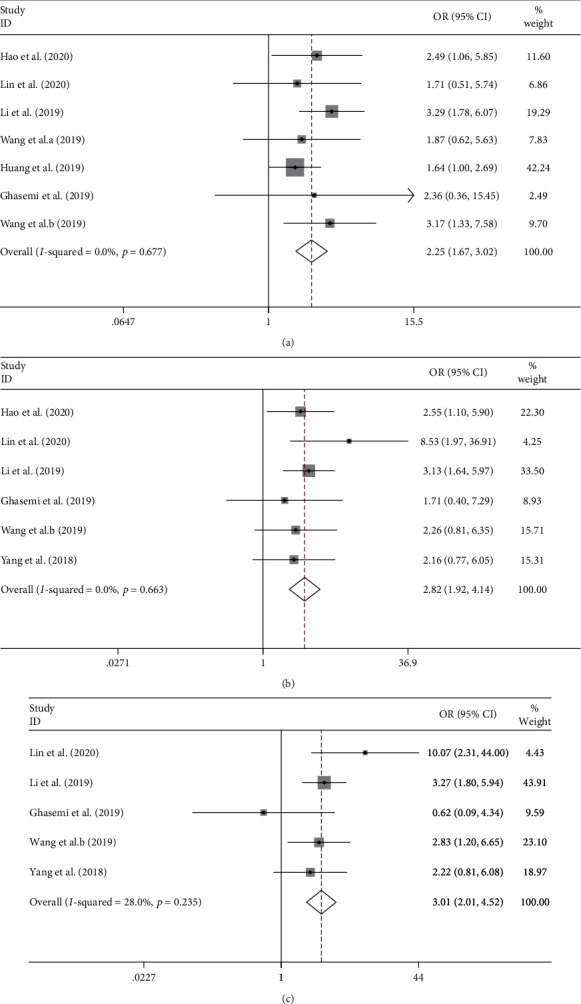
Forest plots of odds ratios (ORs) for the association between circ-ITCH expression and lymph node metastasis (LNM) (a), TNM stage (b), and tumor size (c). The grey squares and horizontal lines denote the study-specific ORs and 95% CIs, respectively. The area of the squares represents the weight (inverse of the variance) of each study. The diamond represents the pooled OR and 95% CI.

As shown in Figure 2, pooled meta-analysis was performed to estimate the relationship between circ-ITCH expression and clinicopathological features of cancers. Since there was no significant heterogeneity among these studies, a fixed-effects model was exploited. The pooled OR with 95% CI indicated that cancer patients with low circ-ITCH expression were more susceptible to develop LNM (OR = 2.25, 95% CI: 1.67-3.02, *p* ≤ 0.01) and advanced TNM stage (OR = 2.82, 95% CI: 1.92-4.14, *p* ≤ 0.01), as well as larger tumor size (OR = 3.01, 95% CI: 2.01-4.52, *p* ≤ 0.01), suggesting that a low circ-ITCH level may serve as an indicator of aggressive clinicopathological features for cancer patients.

### 3.4. Association between circ-ITCH and OS

**Figure 3:**
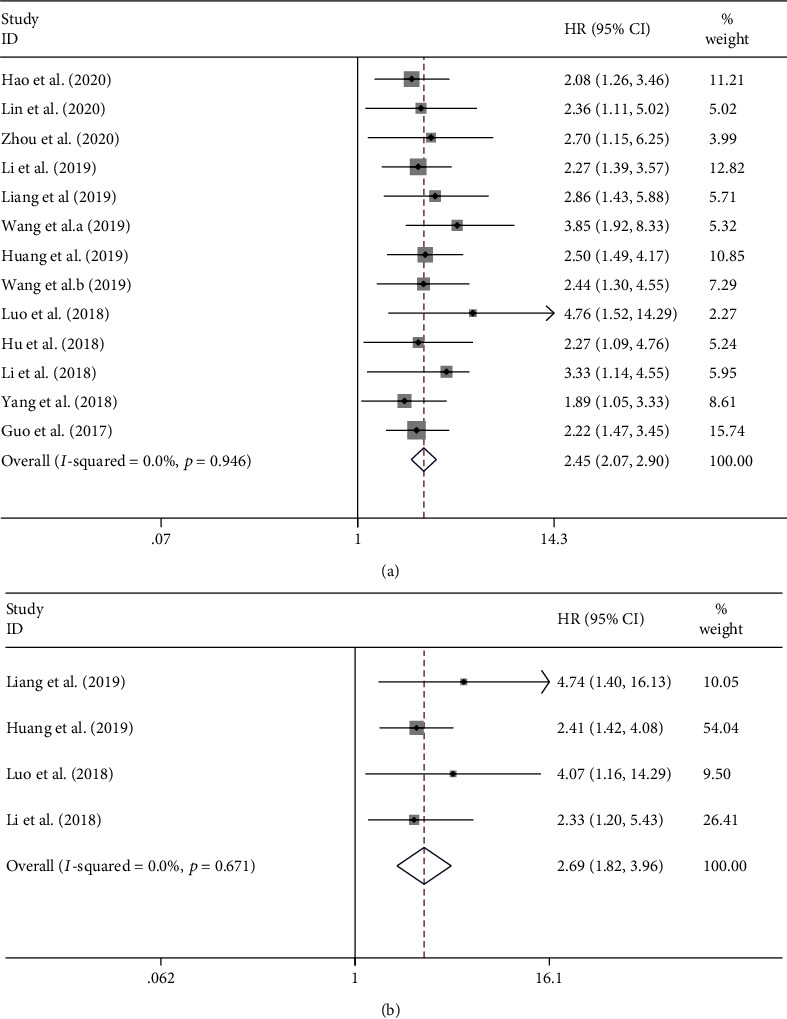
Forest plots of hazard ratios (HRs) for the association between circ-ITCH expression with overall survival (OS) from univariate analysis results (a) and OS from multivariate analysis results (b). The grey squares and horizontal lines denote the study-specific HRs and 95% CIs, respectively. The area of the squares represents the weight (inverse of the variance) of each study. The diamond represents the pooled HR and 95% CI.

On one hand, 13 studies comprising a total number of 1574 patients investigated the association between circ-ITCH expression and OS through univariate analysis. The fixed-effects model was used to assess the pooled HR and its 95% CI since no heterogeneity was found among these studies (*I*^2^ = 0.0%, *p* = 0.946). We found that low circ-ITCH expression was significantly associated with poor OS (HR = 2.45, 95% CI: 2.07–2.90, *p* ≤ 0.01) (Figure 3(a)). Besides, subgroup meta-analysis was also conducted. The results showed that low circ-ITCH expression was a significant prognostic indicator of poor OS for patients with different types of cancers: prostate cancer (HR = 2.88, 95% CI: 1.89-4.39, *p* ≤ 0.01), ovarian cancer (HR = 2.70, 95% CI: 1.82-4.02, *p* ≤ 0.01), and other types of cancers (HR = 2.29, 95% CI: 1.86-2.82, *p* ≤ 0.01). Meanwhile, the significant association between low ITCH expression and unfavorable OS was stable despite the variation of sample size and different cutoff values (Table 2).

On the other hand, 4 studies with a total number of 623 patients investigated the association between circ-ITCH expression and OS through multivariate analysis. Since there was no heterogeneity among these studies (*I*^2^ = 0.0%, *p* = 0.671), the fix-effects model was used to assess the pooled HR and its 95% CI. We found that low circ-ITCH expression was also significantly associated with poor OS (HR = 2.69, 95% CI: 1.82-3.96, *p* ≤ 0.01) (Figure 3(b)).

### 3.5. Sensitive Analysis

**Figure 4:**
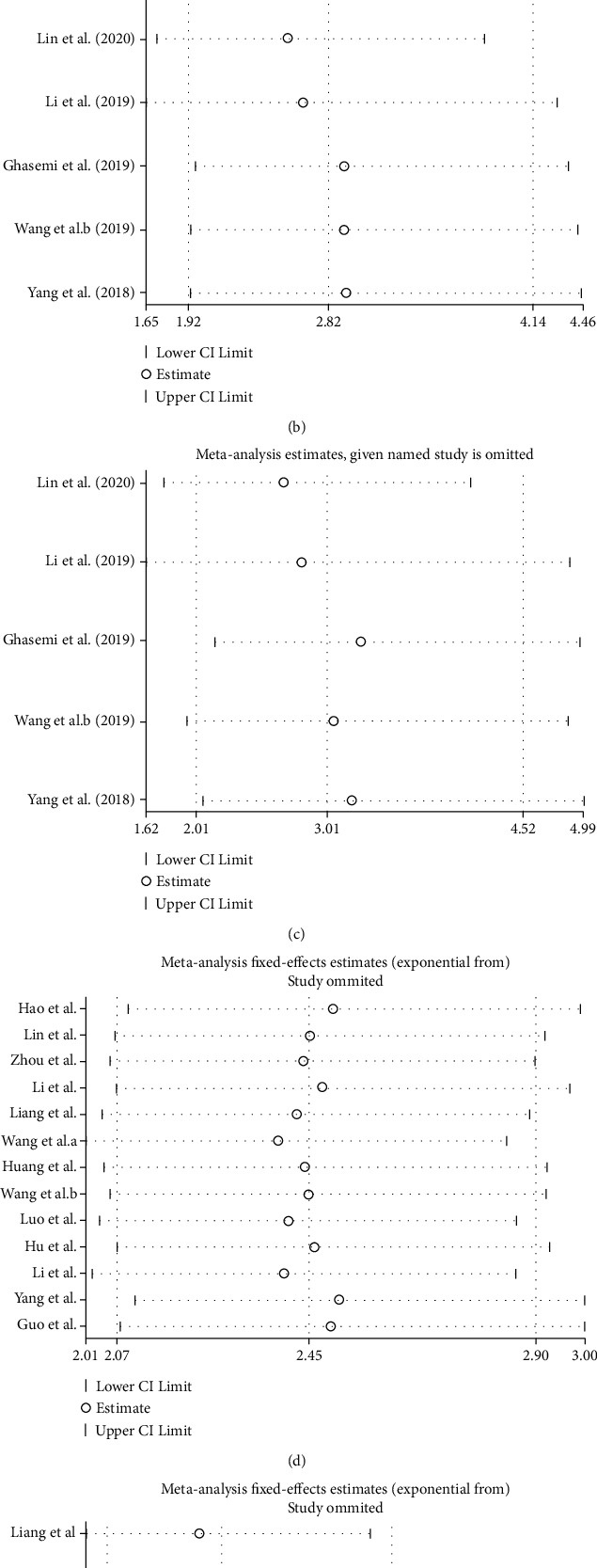
Sensitivity analysis between circ-ITCH expression and lymph node metastasis (LNM) (a), TNM stage (b), tumor size (c), overall survival (OS) via univariate analysis results (d), and OS from multivariate analysis results (e).

To assess the robustness of our results, sensitivity analysis was conducted by omitting each individual included study. As illustrated in Figure 4, removing any of the enrolled studies did not change the overall meta-analysis effect of circ-ITCH on the pooled ORs and HRs, indicating that our findings were relatively stable.

### 3.6. Publication Bias

**Figure 5:**
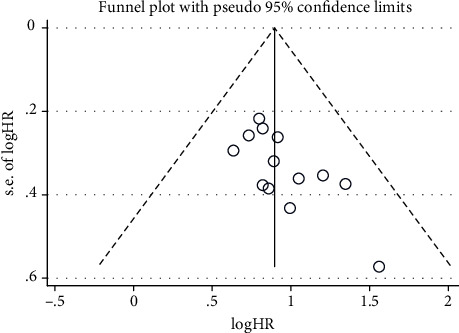
Funnel plots for the meta-analysis with overall survival (OS) from univariate analysis result.

In this meta-analysis, both Begg's and Egger's *p* value tests were used to assess the potential publication bias. No publication bias was found in most analyses, including the studies with LNM (*p* = 0.652, 0.761), TNM stage (*p* = 0.573, 0.890), tumor size (*p* = 0.327, 0.727), and OS (*p* = 0.174, 0.101, multivariate analysis). Publication bias was found in the studies with OS (*p* = 0.038, 0.011, univariate analysis). Besides, the funnel plots of OS from univariate analysis (Figure 5) were largely symmetrical. Therefore, we speculate that most of our meta-analysis results are reliable.

## 4. Discussion

Recently, as increasing studies have demonstrated the participation of circRNAs in carcinogenesis, the potential of circRNA to predict cancer progression has been suggested due to the correlation between their expression and clinicopathological characteristics as well as the outcome of cancers. circ-ITCH was a newly identified circRNA; many studies have investigated the association between circ-ITCH expression and prognosis in cancers. However, the sample sizes of most studies are small. Besides, there is no consensus about the prognostic value of circ-ITCH expression in cancers. Here, we performed this meta-analysis to investigate the clinical and prognostic value of circ-ITCH in cancers.

We included 14 studies with a total of 1604 cancer patients in this meta-analysis. The pooled ORs with their 95% CIs showed that low circ-ITCH expression was significantly associated with larger tumor size, increased LNM, and advanced TNM stage, indicating that low circ-ITCH expression was an indicator of aggressive clinicopathological parameters. Moreover, the pooled HRs with their 95% CIs showed that low circ-ITCH expression was also significantly correlated with poor OS, implying that low circ-ITCH expression may serve as an indicator of unfavorable prognosis of cancers. Since circRNAs lack free 3′ or 5′ tails, they are more resistant to exonuclease RNase R-induced degradation and confer longer half-lives than that of linear mRNAs [2, 38, 39]. Most circRNAs exhibit a half-life longer than 48 h, while an average value of mRNAs is 10 h [40]. Meanwhile, circRNAs are illustrated to be more abundant than their linear isoforms in human cells in a cell type-specific manner [41]; lots of studies have demonstrated that the presence and abundance of circRNAs in different types of cancer cells are distinctive as well [42]. Thus, these remarkable characteristics of circRNAs above could make them serve as detectable biomarkers in diseases [43, 44]. Taken together, circ-ITCH could serve as a biomarker for predicting the progression and outcome of cancers.

Functionally, accumulating evidence has implied a tumor-suppressor role circ-ITCH in diverse cancers. For instance, by acting as a sponge for oncogenic miR-214 and miR-17, circ-ITCH significantly enhances expression of its ITCH linear isoform via competitive interacting with microRNAs, thereby inactivating Wnt/beta-catenin signaling in various cancers [26, 31, 45–47]. Meanwhile, circ-ITCH was also reported to act as ceRNAs of other microRNAs, like microRNA-93-5p and miR-145, to execute its tumor suppressive activity in cervical cancer and ovarian carcinoma, respectively [21, 48]. Additionally, circ-ITCH was further found to inhibit tumorigenesis through other mechanisms rather than acting as a microRNA sponge. In melanoma, circ-ITCH suppresses cancer cell proliferation via impairing glucose uptake of cancer cells [49]. Meanwhile, it has been demonstrated that ectopic expression of circ-ITCH was capable of inhibiting cancer growth in vivo [19, 25], hinting that circ-ITCH might be a potential approach for cancer treatment. The development of RNA-related therapeutics, especially the investigation of strategies to manipulate circRNA levels, may facilitate the circRNA-based therapeutic strategies in the near future [50].

To the best of our knowledge, this is the first meta-analysis to investigate the clinical significance of circ-ITCH in cancer patients. Nevertheless, some limitations of this meta-analysis should be declared. The primary concern is that most of the included studies were conducted based on the population from China; thus, the results should be substantiated by additional studies in the worldwide population. Secondly, publication bias was observed in the studies with OS via univariate analysis. For some studies, the OS results were not available, which may contribute to the publication bias. Based on these limitations above, prospective and well-designed clinical studies with large scale, as well as studies based on other populations beyond China, are still warranted to investigate the role of circ-ITCH in cancer patients.

## 5. Conclusion

In summary, our meta-analysis revealed that low circ-ITCH expression was significantly associated with larger tumor sizes, advanced TNM stage, increased LNM, and poor survival rate in cancers. Therefore, circ-ITCH may serve as a prognostic biomarker and a promising molecular therapy target in cancers.

## Figures and Tables

**Table 1:** Characteristics of included studies in this meta-analysis.

First author (ref.)	Year	Cancer	Country	Sample size	Detection methods	Cutoff value	Clinicopathological features	HR (95% CI)	Data source	Follow-up time (months)	NOS score
Hao et al. [28]	2020	Oral squamous cell carcinoma	China	103	qRT-PCR	Median	①②	OS (U), 2.08 (1.26-3.46)	Curve	Up to 60	8
Lin et al. [24]	2020	Ovarian cancer	China	45	qRT-PCR	Median	①②③	OS (U), 2.36 (1.11-5.02)	Curve	Up to 60	8
Zhou et al. [32]	2020	Multiple myeloma	China	92	qRT-PCR	Median	NA	OS (U), 2.70 (1.15-6.25)	Direct	Median 24.5	7
Li et al. [27]	2019	Lung cancer	China	190	qRT-PCR	Median	①②③	OS (U), 2.27 (1.39-3.57)	Curve	Up to 36	8
Liang et al. [23]	2019	Ovarian cancer	China	122	qRT-PCR	NA	NA	OS (U), 2.86 (1.43-5.88) OS (M), 4.74 (1.40-16.13)	Direct	Median 30	6
Wang et al.^a^ [19]	2019	Prostate cancer	China	52	qRT-PCR	Median	①	OS (U), 3.85 (1.92-8.33)	Curve	Up to 70	8
Huang et al. [20]	2019	Prostate cancer	China	364	qRT-PCR	Median	①	OS (U), 2.50 (1.49-4.17) OS (M), 2.41 (1.42-4.08)	Direct	Up to 60	8
Ghasemi et al. [29]	2019	Gastric cancer	Iran	30	qRT-PCR	Median	①②③	NA	NA	NA	7
Wang et al.^b^ [26]	2019	Breast cancer	China	91	qRT-PCR	Median	①②③	OS (U), 2.44 (1.30-4.55)	Curve	Up to 100	8
Luo et al. [22]	2018	Ovarian cancer	China	77	qRT-PCR	Median	NA	OS (U), 4.76 (1.52-14.29) OS (M), 4.07 (1.16-14.29)	Direct	Median 28	7
Hu et al. [21]	2018	Ovarian cancer	China	20	qRT-PCR	NA	NA	OS (U), 2.27 (1.09-4.76)	Curve	Up to 140	6
Li et al. [31]	2018	Glioma	China	60	qRT-PCR	0.36^∗^	NA	OS (U), 3.33 (1.14-4.55) OS (M), 2.33 (1.20-5.43)	Direct	Up to 80	7
Yang et al. [25]	2018	Bladder cancer	China	70	qRT-PCR	NA	②③	OS (U), 1.89 (1.05-3.33)	Curve	1-60	7
Guo et al. [30]	2017	Hepatocellular carcinoma	China	288	qRT-PCR	Median	NA	OS (U), 2.22 (1.47-3.45)	Direct	Up to 90	7

NA: not available; qRT-PCR: quantitative reverse transcription-polymerase chain reaction; ①: lymph node metastasis (LNM); ②: TNM stage; ③: tumor size; OS: overall survival; U: univariate analysis; M: multivariate analysis; curve: Kaplan-Meier curve; ^∗^the cutoff value of this study is 0.36 which was established by the receiver operating characteristic (ROC) analysis.

**Table 2:** Stratification analysis for the meta-analysis with overall survival (OS) in patients with cancers.

Subgroup	No. of studies	No. of patients	Pooled HR (95% CI)	Heterogeneity	
				*I* ^2^ (%)	*p* value
Cancer type					
Prostate cancer	2	416	2.88 (1.89-4.39)	0.0	0.345
Ovarian cancer	4	264	2.70 (1.82-4.02)	0.0	0.720
Others	7	894	2.29 (1.86-2.82)	0.0	0.928
Cutoff value					
Median	9	1302	2.45 (2.01-2.98)	0.0	0.894
Others	4	272	2.46 (1.76-3.43)	0.0	0.622
Sample size					
>100	5	1067	2.31 (1.85-2.90)	0.0	0.959
<100	8	507	2.64 (2.04-3.41)	0.0	0.766

## Data Availability

The data supporting this meta-analysis are from previously reported studies that have been cited. The processed data are available from the corresponding author upon request.

## References

[1] Patop I. L., Wust S., Kadener S. (2019). Past, present, and future of circRNAs. *The EMBO Journal*.

[2] Memczak S., Jens M., Elefsinioti A. (2013). Circular RNAs are a large class of animal RNAs with regulatory potency. *Nature*.

[3] Wilusz J. E. (2018). A 360 degrees view of circular RNAs: from biogenesis to functions. *Wiley interdisciplinary reviews RNA*.

[4] Han D., Li J., Wang H. (2017). Circular RNA circMTO1 acts as the sponge of microRNA-9 to suppress hepatocellular carcinoma progression. *Hepatology*.

[5] Hansen T. B., Wiklund E. D., Bramsen J. B., Villadsen S. B., Statham A. L., Clark S. J. (2011). miRNA-dependent gene silencing involving Ago2-mediated cleavage of a circular antisense RNA. *The EMBO Journal*.

[6] Li Z., Huang C., Bao C. (2015). Exon-intron circular RNAs regulate transcription in the nucleus. *Nature Structural & Molecular Biology*.

[7] Pamudurti N. R., Bartok O., Jens M. (2017). Translation of circRNAs. *Molecular Cell*.

[8] Peng L., Yuan X. Q., Li G. C. (2015). The emerging landscape of circular RNA ciRS-7 in cancer (review). *Oncology Reports*.

[9] Weng W., Wei Q., Toden S. (2017). Circular RNA ciRS-7—a promising prognostic biomarker and a potential therapeutic target in colorectal cancer. *Clinical Cancer Research: An Official Journal of the American Association for Cancer Research*.

[10] Li R. C., Ke S., Meng F. K. (2018). ciRS-7 promotes growth and metastasis of esophageal squamous cell carcinoma via regulation of miR-7/HOXB13. *Cell Death & Disease*.

[11] Huang H., Wei L., Qin T., Yang N., Li Z., Xu Z. (2019). Circular RNA ci RS-7 triggers the migration and invasion of esophageal squamous cell carcinoma via mi R-7/KLF4 and NF-kappa B signals. *Cancer Biology & Therapy*.

[12] Zhong Z., Huang M., Lv M. (2017). Circular RNA MYLK as a competing endogenous RNA promotes bladder cancer progression through modulating VEGFA/VEGFR2 signaling pathway. *Cancer Letters*.

[13] Kun-Peng Z., Xiao-Long M., Chun-Lin Z. (2018). Overexpressed circPVT1, a potential new circular RNA biomarker, contributes to doxorubicin and cisplatin resistance of osteosarcoma cells by regulating ABCB1. *International Journal of Biological Sciences*.

[14] Chen Z., Zhang L., Han G. (2018). A meta-analysis of the diagnostic accuracy of circular RNAs in digestive system malignancy. *Cellular Physiology and Biochemistry: International Journal of Experimental Cellular Physiology, Biochemistry, and Pharmacology*.

[15] Huang X., Zhang W., Shao Z. (2019). Prognostic and diagnostic significance of circRNAs expression in hepatocellular carcinoma patients: a meta-analysis. *Cancer Medicine*.

[16] Li F., Huang Q., Gong Z., Wang H., Chen J. (2019). Diagnostic and prognostic roles of circ-SHPRH for solid cancers: a meta-analysis. *Oncotargets and Therapy*.

[17] Li C., Zhang L., Meng G. (2019). Circular RNAs: pivotal molecular regulators and novel diagnostic and prognostic biomarkers in non-small cell lung cancer. *Journal of Cancer Research and Clinical Oncology*.

[18] Li Y., Ge Y. Z., Xu L., Jia R. (2019). Circular RNA ITCH: a novel tumor suppressor in multiple cancers. *Technology in Cancer Research & Treatment*.

[19] Wang X., Wang R., Wu Z., Bai P. (2019). Circular RNA ITCH suppressed prostate cancer progression by increasing HOXB13 expression via spongy miR-17-5p. *Cancer Cell International*.

[20] Huang E., Chen X., Yuan Y. (2019). Downregulated circular RNA itchy E3 ubiquitin protein ligase correlates with advanced pathologic T stage, high lymph node metastasis risk and poor survivals in prostate cancer patients. *Artificial cells, nanomedicine, and biotechnology*.

[21] Hu J. H., Wang L., Chen J. M. (2018). The circular RNA circ-ITCH suppresses ovarian carcinoma progression through targeting miR-145/RASA1 signaling. *Biochemical and Biophysical Research Communications*.

[22] Luo L., Gao Y., Sun X. (2018). circ-ITCH correlates with small tumor size, decreased FIGO stage and prolonged overall survival, and it inhibits cells proliferation while promotes cells apoptosis in epithelial ovarian cancer. *Cancer biomarkers: section A of Disease markers*.

[23] Liang Y. H. (2019). The correlation between circ-itch expression level and the clinicopathological features and prognosis of patients with epithelial ovarian cancer and its effect on the proliferation and apoptosis of ovarian cancer cells. *Journal of Fujian Medical University*.

[24] Lin C., Xu X., Yang Q., Liang L., Qiao S. (2020). Circular RNA ITCH suppresses proliferation, invasion, and glycolysis of ovarian cancer cells by up-regulating CDH1 via sponging miR-106a. *Cancer Cell International*.

[25] Yang C., Yuan W., Yang X. (2018). Circular RNA circ-ITCH inhibits bladder cancer progression by sponging miR-17/miR-224 and regulating p21, PTEN expression. *Molecular Cancer*.

[26] Wang S. T., Liu L. B., Li X. M. (2019). circ-ITCH regulates triple-negative breast cancer progression through the wnt/*β*-catenin pathway. *Neoplasma*.

[27] Li Z., Guo X., Gao S. (2019). circ-ITCH correlates with less advanced tumor features as well as prolonged survival, and it inhibits cells proliferation but promotes apoptosis in non-small cell lung cancer. *Translational Cancer Research*.

[28] Hao C., Wangzhou K., Liang Z. (2020). Circular RNA ITCH suppresses cell proliferation but induces apoptosis in oral squamous cell carcinoma by regulating mi R-421/PDCD4 axis. *Cancer Management and Research*.

[29] Ghasemi S., Emadi-Baygi M., Nikpour P. (2019). Down-regulation of circular RNAITCH and circHIPK3 in gastric cancer tissues. *Turkish journal of medical sciences*.

[30] Guo W., Zhang J., Zhang D. (2017). Polymorphisms and expression pattern of circular RNA circ-ITCH contributes to the carcinogenesis of hepatocellular carcinoma. *Oncotarget*.

[31] Li F., Ma K., Sun M. H., Shi S. (2018). Identification of the tumor-suppressive function of circular RNA ITCH in glioma cells through sponging miR-214 and promoting linear ITCH expression. *American Journal of Translational Research*.

[32] Zhou H., Zhang J., Chen B. (2020). Potential of circular RNA itchy E3 ubiquitin protein ligase as a biomarker and treatment target for multiple myeloma. *Translational Cancer Research*.

[33] Moher D., Liberati A., Tetzlaff J., Altman D. G., Group P (2009). Preferred reporting items for systematic reviews and meta-analyses: the PRISMA statement. *PLoS Medicine*.

[34] Tierney J. F., Stewart L. A., Ghersi D., Burdett S., Sydes M. R. (2007). Practical methods for incorporating summary time-to-event data into meta-analysis. *Trials*.

[35] Stang A. (2010). Critical evaluation of the Newcastle-Ottawa scale for the assessment of the quality of nonrandomized studies in meta-analyses. *European Journal of Epidemiology*.

[36] Egger M., Davey Smith G., Schneider M., Minder C. (1997). Bias in meta-analysis detected by a simple, graphical test. *BMJ*.

[37] Begg C. B., Mazumdar M. (1994). Operating characteristics of a rank correlation test for publication bias. *Biometrics*.

[38] Chen L. L. (2016). The biogenesis and emerging roles of circular RNAs. *Nature Reviews Molecular Cell Biology*.

[39] Enuka Y., Lauriola M., Feldman M. E., Sas-Chen A., Ulitsky I., Yarden Y. (2016). Circular RNAs are long-lived and display only minimal early alterations in response to a growth factor. *Nucleic Acids Research*.

[40] Jeck W. R., Sharpless N. E. (2014). Detecting and characterizing circular RNAs. *Nature Biotechnology*.

[41] Glazar P., Papavasileiou P., Rajewsky N. (2014). circBase: a database for circular RNAs. *RNA*.

[42] Salzman J., Chen R. E., Olsen M. N., Wang P. L., Brown P. O. (2013). Cell-type specific features of circular RNA expression. *PLoS Genetics*.

[43] Meng S., Zhou H., Feng Z. (2017). CircRNA: functions and properties of a novel potential biomarker for cancer. *Molecular Cancer*.

[44] Zhang Z., Yang T., Xiao J. (2018). Circular RNAs: promising biomarkers for human diseases. *eBioMedicine*.

[45] Wan L., Zhang L., Fan K., Cheng Z. X., Sun Q. C., Wang J. J. (2016). Circular RNA-ITCH suppresses lung cancer proliferation via inhibiting the Wnt/beta-catenin pathway.

[46] Li F., Zhang L., Li W. (2015). Circular RNA ITCH has inhibitory effect on ESCC by suppressing the Wnt/*β*-catenin pathway. *Oncotarget*.

[47] Huang G., Zhu H., Shi Y., Wu W., Cai H., Chen X. (2015). cir-ITCH plays an inhibitory role in colorectal cancer by regulating the Wnt/*β*-catenin pathway. *Plo S one*.

[48] Li J., Guo R., Liu Q., Sun J., Wang H. (2020). Circular RNA Circ-ITCH inhibits the malignant behaviors of cervical cancer by microRNA-93-5p/FOXK2 axis. *Reproductive sciences*.

[49] Lin Q., Jiang H., Lin D. (2019). Circular RNA ITCH downregulates GLUT1 and suppresses glucose uptake in melanoma to inhibit cancer cell proliferation. *The Journal of Dermatological Treatment*.

[50] Santer L., Bar C., Thum T. (2019). Circular RNAs: a novel class of functional RNA molecules with a therapeutic perspective. *Molecular therapy: the journal of the American Society of Gene Therapy*.

